# Relative Impact of Birth Weight and Early Growth on Neonatal Mortality in Puppies

**DOI:** 10.3390/ani13121928

**Published:** 2023-06-09

**Authors:** Amélie Mugnier, Virginie Gaillard, Sylvie Chastant

**Affiliations:** 1 NeoCare, Université de Toulouse, ENVT, 31300 Toulouse, France; amelie.mugnier@envt.fr (A.M.); sylvie.chastant@envt.fr (S.C.); 2 Royal Canin Research Center, 30470 Aimargues, France

**Keywords:** puppy, low birth weight, early growth, neonate, mortality, threshold, CART

## Abstract

**Simple Summary:**

The mortality rate in the first two months of life is high in canine species, estimated at about 10% of live-born puppies, and can certainly be improved. Early identification of neonates at higher risk of mortality is required. Using data collected from 8550 puppies shared on a voluntary basis by 127 French breeding kennels, we explored the early growth of puppies as well as its interconnections with birth weight and mortality within the first two months. Low-birth-weight puppies were found to grow less than others over the first days of life but more later (compensatory growth). Thresholds for growth rates allowing the identification of puppies at higher risk of mortality during their first two months of life were established and will be useful to identify puppies with insufficient early growth to improve their chances of survival.

**Abstract:**

Puppy survival during their first weeks of life can be improved, and early detection of puppies with increased mortality risk is one of the keys to success. In the canine species, the few studies on this subject focused on birth weight, which reflects intrauterine growth. The present work aimed to explore the interconnections between birth weight, early growth and survival until two months of life in the canine species. In total, data from 8550 puppies born in 127 French breeding kennels were analysed. Five different growth rates were calculated to reflect the growth of puppies during their first week of life. Low-birth-weight puppies had lower growth than normal-birth-weight puppies over the first two days of life but higher growth rates thereafter. Growth-rate thresholds allowing the identification of puppies at higher risk of mortality during their first two months of life were lower for low-birth-weight puppies. These thresholds will help breeders and veterinarians to identify puppies at risk with particular needs for monitoring and nursing to improve their chances of survival.

## 1. Introduction

Canine newborns face a high mortality rate (approximately 10% of live-born puppies) during their first two months of life, i.e., during which they live with their mother at the breeder’s [1,2,3,4]. Such neonatal losses are major issues regarding animal welfare and kennel economic viability. Various strategies can be considered to reduce puppy mortality during the first two months of life. One path is to prevent the birth of low-birth-weight (LBW) puppies since in dogs, as in many species, LBW has been identified as a major risk factor for morbidity and mortality during the first weeks of life [5,6,7,8,9]. Low birth weight is generally the result of intrauterine growth restriction, with its aetiology poorly understood and insufficiently investigated in the canine species. Due to the current paucity of objective guidelines to mitigate this intrauterine growth restriction, a more plausible interim solution appears to be to improve the survival rates of LBW puppies. In piglets, some studies report the benefits of interventions, such as monitoring and specific care of LBW animals [10,11,12]. The high mortality rate of LBW newborns is associated with limited energy reserves, hypoglycaemia and hypothermia [13,14,15,16,17]. Nutritional management, i.e., energy supplementation or controlled suckling, demonstrated positive effects on survival [10,18,19,20] and thus helped to counteract the deleterious effect of reduced intrauterine growth. Early growth monitoring has also been proposed to help piglet management, e.g., using early growth rate as a proxy of the amount of colostrum ingested, which is crucial for the survival of newborns [21,22]. Thus, it would be of practical interest to assess whether growth over the first days of life is associated with the mortality rate in LBW newborn puppies.

The first objective of this study, based on data collected on a national scale, was to describe and compare early growth rates for low- and normal-birth-weight puppies. Then, this study explored the association between early growth (during the first week of life) and mortality rate over the first two months of life, considering the birth-weight category. Finally, specific growth-rate thresholds have been determined to allow breeders and veterinarians to assess the growth of puppies during their first days of life and identify those at higher risk of mortality over the first two months of life. 

## 2. Materials and Methods

### 2.1. Study Population

Data were collected through a questionnaire distributed to French dog breeders from 2015 to 2019 using direct mailings, Facebook^®^ messages, during canine exhibitions and via various dog breed associations [9]. The completion of the questionnaire was voluntary. The recorded data included information about the litter (date of birth, breed, litter size at birth), dam (identity and age at whelping) and puppy (sex, daily weights during the first week of life and eventually death declaration in case of death during the first two months after birth). Weights were measured by the breeders using their own scales. The information collected was transferred anonymously into an Excel table (Microsoft Corporation, Redmond, Washington, DC, USA) for analysis with the breeders’ consent.

Among all the data collected, only purebred puppies with a known status (dead or alive) at two months of age, with birth weight provided and born beyond the year 2000 were considered for the present study.

### 2.2. Data Management

Five growth rates over the first week of life were calculated: between birth (Day 0) and Day 1 (GR 0–1), between Day 1 and Day 2 (GR 1–2), between birth and Day 2 (GR 0–2), between Day 2 and Day 7 (GR 2–7) and between birth and Day 7 (GR 0–7). Growth rates were calculated as: GR (x–y) = [(weight at Day y − weight at Day x) ÷ weight at Day x] × 100. Prior to analysis, puppies with extreme values were removed, i.e., with values higher than the 75th centile plus 1.5 times the interquartile range (IQR) or lower than the 25th centile minus 1.5 times the IQR (Tukey’s method). 

Only puppies belonging to a breed represented by at least 100 individuals were included in the analysis.

### 2.3. Birth-Weight Classification

Puppies were categorized as low or normal birth weight (LBW or NBW) based on their status at 2 months (dead or alive), using thresholds defined by classification and regression tree (CART) analysis. To deal with the variability of birth weight depending on the breed [1,23], thresholds were breed-specific. For each breed, CART was used to assess the ability of birth weight to discriminate between the survivors and the puppies that would die over their two first months of life. CART is a statistical technique based on the recursive partitioning method well suited to define clinical decision rules [24]. Briefly, it consists of repeatedly partitioning the data into subgroups leading to the construction of a decision tree [25,26]. The method provides a rule (here, a cut-off value) used for predicting the outcome variable (here, dead or alive status at 2 months). To prevent the model from overfitting the dataset, any node defining a population with less than 20 subjects was forced to become a terminal node. The maximal depth of the trees was fixed at one (identification of a single threshold). The Gini index was used as the splitting method, and 10-fold cross-validation repeated 5 times was used as the method for testing the trees obtained. RMSE was used to select the optimal model using the smallest value.

### 2.4. Early Growth Analysis

After a general description of the five growth-rate distributions, means were compared between NBW and LBW using univariate analyses of variance (ANOVA) after testing the assumptions. Then, CART analysis, following the same procedure as described above, was used to assess the ability of growth rate to discriminate between the survivors and the puppies that would die during the two first months of life. NBW and LBW were analysed separately, and cut-off values were determined for each growth rate (GR0–1, GR1–2, GR 0–2, GR 2–7 and GR 0–7).

### 2.5. Ranking of Weight-Related Puppy Mortality Risk Factors

In the last part of the analysis, the CART procedure, using the same method as described above, allowed identifying weight-related variables with the highest discriminatory power to differentiate between puppies dying before two months of age and those still alive at the end of the period considered. The procedure was carried out three times: for puppies alive on Day 1, then those alive on Day 2 and finally those alive on Day 7. On Day 1, birth-weight category (LBW or NBW) and GR 0–1 were considered. On Day 2, birth-weight category, GR 0–1 and GR 1–2 were considered. Finally, on Day 7, birth-weight category, GR 0–1, GR 1–2 and GR 2–7 were considered. The relative discriminatory power of each variable included in the analysis was calculated in order to rank weight-related mortality risk factors on Day 1, 2 and 7. 

All the statistical analyses were performed using R software, version 4.2.1 [27], and the following packages: *ggplot2* [28] for data visualisation, *dplyr* [29] and *tidyr* [30] for data manipulation and *rpart* [31] and *caret* [32] to perform CART analyses. A value of *p* < 0.05 was considered statistically significant, and statistical uncertainty was assessed by calculating 95% confidence intervals (95 CI).

## 3. Results

### 3.1. Population Characteristics

**Figure 1:**
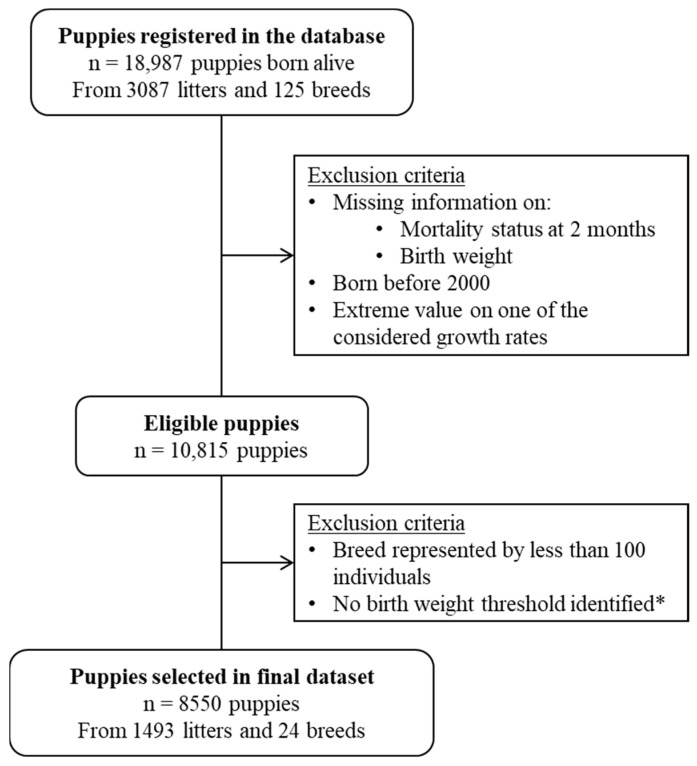
Flow diagram describing the data-selection process. * The thresholds were established by breed using CART analysis in order to separate low- and normal-birth-weight puppies according to their mortality risk during the first two months of life.

Data from a total of 8550 live-born puppies from 24 breeds, 1493 litters, and belonging to 127 French breeding kennels were included in this study (Figure 1). Litters were born between 2000 and 2019 (71% after 2010). Among the 24 breeds included, 11 were in the top-twenty breeds owned in France, according to the French Kennel Club [33]. The number of puppies included per breed ranged from 105 for the Greyhound to 1802 for the Labrador Retriever (median: 228). The global mean litter size (alive at birth) was 7.5 (SD: 2.6) puppies and the sex ratio was calculated at 1.0 (4235 males vs. 4287 females). Birth-weight values ranged from 56 g (a Pomeranian puppy) to 940 g (a Newfoundland puppy), with a median of 380 g (IQR: 260–470).

Among these 8550 puppies, based on their 0–2 months mortality and CART analysis, 1291 (15%) were classified as LBW and 7259 (85%) as NBW. Birth-weight-threshold values by breed are presented in Supplementary data (Table S1).

### 3.2. Description of Mortality

A total of 8.9% (758/8550; 95% CI: 8.3–9.5) of live-born puppies died during the first two months of life, with most of the deaths (88%, 669/758) occurring during the neonatal period (between birth and Day 21).

**Figure 2:**
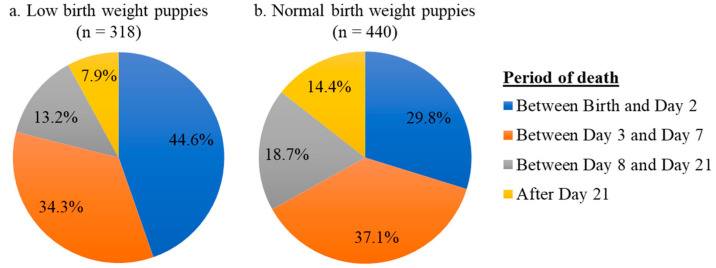
Pie charts describing the distribution of deaths depending on birth-weight category.

CART analysis allowed the creation of two groups of puppies based on their status at 2 months (dead or alive). The overall mortality rate during the first two months of life was thus higher for LBW than for NBW puppies: 24.6% (95% CI: 22.3–27.1) vs. 6.1% (95% CI: 5.5–6.6), respectively. In addition, the time distribution of deaths was also different depending on the birth-weight category (Figure 2): LBW puppies died significantly more frequently during the first two days of life and significantly less after the third week of life than NBW puppies (z-test for equality of proportions without correction, *p* < 0.001).

### 3.3. Description of Early Growth

Overall growth rates, as well as values for LBW and NBW puppies, are described in Table 1. One-way ANOVAs revealed that there were statistically significant differences between NBW and LBW puppies for all the growth rates considered (Table 1). Growth rates were lower for LBW than for NBW during the first two days of life (F(1, 6501) = 7.96, *p* < 0.01 for GR 0–1; F(1, 6246) = 40.37, *p* < 0.001 for GR 1–2 and F(1, 7387) = 15.86, *p* < 0.001 for GR 0–2). Conversely, later than Day 2, LBW presented higher growth rates than NBW; for GR 2–7 (F(1, 6335) = 9.32, *p* < 0.01) and also for GR 0–7 (F(1, 6809) = 5.21, *p* < 0.05). 

### 3.4. Identification of Early-Growth Thresholds

For the five growth rates considered, CART analysis was able to determine thresholds discriminating two categories of puppies with different mortality risks, suggesting a link between early growth and mortality over the first two months after birth. Such a link was evidenced both for LBW and for NBW puppies, but growth-rate thresholds differ between the two categories (Table 2). 

### 3.5. Weight-Related Risk Factors Associated with Puppy Mortality

In the last part of the analysis, the relative importance of weight-related parameters over the first week after birth on mortality risk over the first two months was assessed using CART analyses. The method was applied three times by considering puppies alive on Day 1, then puppies alive on Day 2 and, finally, puppies alive on Day 7. Depending on the age at assessment, different growth rates were included in the CART analysis. Their relative importance, as well as that of birth weight, was assessed without regard to the timing of the measurements (Table 3).

**Figure 3:**
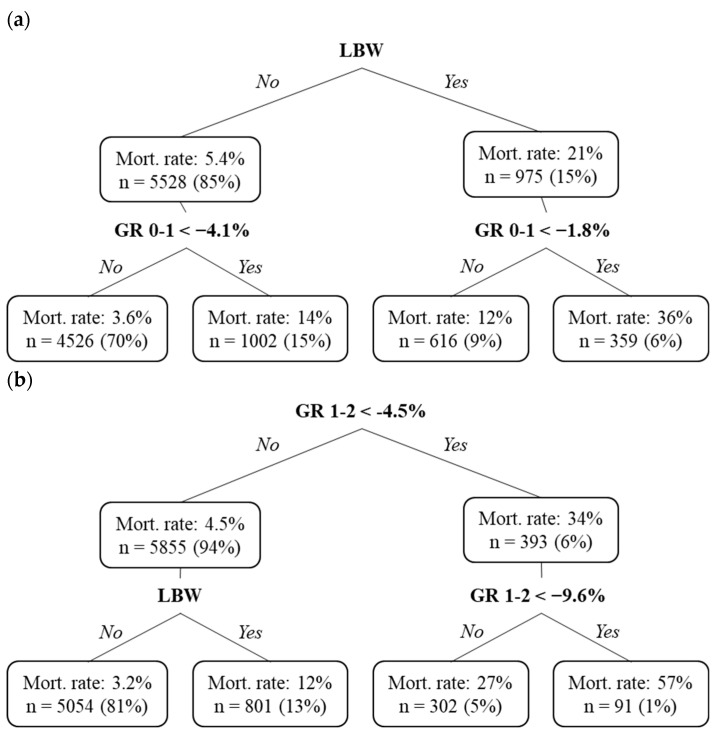

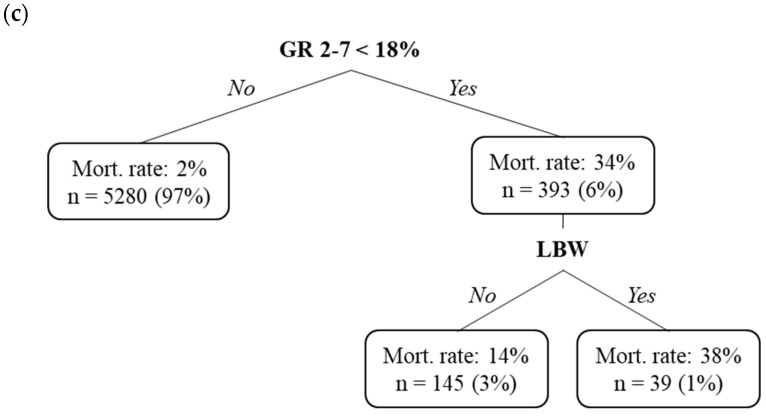
Early risk factors for puppy mortality during the first two months of life: classification trees at (**a**) Day 1, (**b**) Day 2 and (**c**) Day 7. In each box, Mort. rate indicates the mortality rate until 2 months of age in the group considered, *n*, the number of puppies and the percentage of total population is mentioned between brackets. (**a**) Classification tree considering 6503 puppies alive at Day 1, of which 7.8% will die before 2 months of life. (**b**) Classification tree considering 6248 puppies alive at Day 2, of which 6.3% will die before 2 months of life. (**c**) Classification tree considering 5464 puppies alive at Day 7, of which 2.6% will die before 2 months of life.

The relative importance of the birth-weight category in distinguishing puppies alive or not at two months of age compared to one of the growth rates included in the CART analysis was high on Day 7, moderate on Day 2 and not significant on Day 1 (Table 3). On Day 1 and Day 2, growth rates over the first days were the most important predictive variables. Figure 3 presents three classification trees used to discriminate survivors from puppies dying during the first two months of life based on weight-related variables assessed between birth and seven days of life.

## 4. Discussion

In the scientific literature, different time windows have been used to describe postnatal mortality in the canine species (0–7 days [34], 0–21 days [3,6], 0–1 month [35], 0–6 weeks [4]). In our study, we chose to assess mortality between birth and two months of age, i.e., over the period managed by breeders until adoption (2 months is the minimum legal age of sale for a puppy in France). After the exclusion of stillborn puppies, the mortality rate over the first two months of life in our population (8.9%; 95% CI: 8.3–9.5) was slightly higher than that observed over the same period in previous studies (6% in [2] and 7.5% in [3]). The distribution of mortality cases (Figure 2) indicates that the vast majority of deaths occur within the first week of life, especially for LBW puppies. As the objective of the present study was to identify growth-rate thresholds for the appropriate monitoring and management of LBW puppies, we focused on growth indicators during the first week of life.

### 4.1. Early Growth

Overall, the growth rate in our population over the first week was consistent with previous studies in dogs [3,36]. During the first two days of life, mean growth rates were positive (Table 1), meaning that most puppies gained weight during their early life. This is contrary to the study of Bigliardi et al. [37], conducted with a standardised weighing method in Boxers born in a single breeding kennel. Indeed, they reported a “physiological” weight loss over the first three days of life (−11.3%), explained by the expulsion of urine and meconium. This is similar to what happens in human neonatology, where newborns take about three days to retrieve birth weight [38]. More studies on various breeds are needed to assess what proportion of puppies gain or lose weight during the first days of life.

Even LBW puppies exhibit positive growth rates (Table 1). All growth rates were found to differ depending on the birth-weight category (LBW vs. NBW, Table 1), which is slightly different from an earlier study that described no influence of birth weight on GR 0–2 [6].

Lower growth rates during the first two days in LBW compared with NBW could be explained by their reduced capacity to adapt to extrauterine life [15,39]. This is confirmed by a higher mortality rate of LBW during the first two days of life (Figure 2). Between Day 2 and Day 7, our work found higher growth rates in LBW puppies compared with NBW puppies. This result suggests that LBW puppies experience the onset of compensatory growth started within the first week of life. This phenomenon of compensatory growth of LBW newborns has been widely described in humans and rodents [40,41,42,43]. Conversely, piglets with higher birth weights were associated with a higher body-weight gain during the suckling period without any compensatory growth in LBW [7,44,45]. This difference may be explained by limited access to milk [46], as LBW piglets have to compete against heavier siblings to access the teats. In our study, the amount of assistance provided by breeders to neonates was not recorded due to the retrospective nature of this research. Neither the practice of controlled suckling nor bottle feeding supplementation, if any, was known, meaning that the growth observed in the present work may be partly assisted and not fully spontaneous. However, working in vivo on strictly spontaneous growth without any assistance, even if needed, would be unethical.

### 4.2. Growth-Rate Thresholds

Weighing meets all the conditions to be fulfilled directly by breeders in everyday practice. Indeed, although it requires specific equipment (scales with appropriate accuracy), the need for investment is low, the practice is simple, requiring no specific skills, and the result is readily available. However, there is a lack of guidelines for assessing the adequacy of puppy growth. A threshold for a growth rate between birth and Day 2 based on the risk of neonatal mortality (Day 0–Day 21) was previously defined at −4% [6]. In another study, nearly half of the puppies gained weight from birth (5–10% daily between Day 0 and Day 21) [47]. In the present work, growth-rate thresholds were determined based on the risk of mortality over the first two months of life. Calculations were performed separately for LBW and NBW puppies for five periods. Such thresholds were set to discriminate between puppies dying or not before the age of two months. As thresholds between birth and Day 2 were lower for NBW than for LBW (Table 2), NBW puppies seem more tolerant to early postnatal growth reduction (Table 2): higher weight loss will result in a lower risk of mortality later on. Since it has been possible to determine the relevant growth-rate thresholds for both LBW and NBW puppies, growth should be monitored in all puppies. The thresholds provided by the present study will allow close monitoring of newborn puppies over the first week after birth. Further studies are warranted to assess whether thresholds could be refined by considering other factors such as the breed size (or even breed), puppy’s sex, litter size and environmental conditions. In addition, as they were established using French data, extrapolation to other countries should be performed with caution due to differences in breed lineages.

### 4.3. Relative Importance of the Different Weight-Related Parameters

Since we evidenced the influence of birth weight together with growth rates over several periods on mortality rate, the final part of the analysis aimed at ranking the weight-related criteria according to their ability to discriminate puppies surviving from those dying before two months of age. The results revealed that growth rate could help to reduce but not totally reverse the deleterious impact of the birth-weight category. Interestingly, birth weight was important to discriminate surviving puppies not only for mortality during the first days of life but also after the first week. Indeed, the birth-weight category was the most discriminant criterion for puppies alive at Day 7 to assess their chances of survival until two months of age (Table 3). This suggests that birth weight is not only related to very early neonatal mortality but can also impact later life, in this case, with a midterm impact. This result is in line with that of Gaillard et al. [48] and Mugnier et al. [49], suggesting long-term consequences of reduced intrauterine growth in the canine species. 

## 5. Conclusions

This study justifies and provides practical guidelines for the measurement of the daily growth rate of puppies during their first week of life. The cut-off values calculated in our study provide an easy tool for detecting and nursing puppies at increased risk of mortality. They could be relevant for breeders and veterinarians to objectively identify early puppies requiring particular attention. The next step towards improving canine health and welfare could be to define optimal growth by considering together the short- and long-term impacts of early growth. Indeed, optimal early growth probably does not mean maximum growth because of the potential risk of being overweight in adulthood. Cohort studies are needed to determine lower (risk of mortality) and higher (risk of overweight) thresholds throughout early growth in the canine species.

## Figures and Tables

**Table 1:** Mean growth rates and comparison by birth-weight categories.

	Mean Growth Rates, % (±SD)			*p* ^1^
	All Puppies	LBW	NBW	
**GR 0–1**	1.6 (±6.4) (*n* = 6503)	1.1 (±7.1) (*n* = 975)	1.7 (±6.2) (*n* = 5528)	**
**GR 1–2**	6 (±6.6) (*n* = 6248)	4.7 (±7.3) (*n* = 898)	6.2 (±6.4) (*n* = 5350)	***
**GR 0–2**	7.3 (±10.5) (*n* = 7389)	6.1 (±11.6) (*n* = 1038)	7.5 (±10.3) (*n* = 6351)	***
**GR 2–7**	54.3 (±18.4) (*n* = 6337)	56.1 (±20.9) (*n* = 818)	54 (±18) (*n* = 5519)	**
**GR 0–7**	67.1 (±28.7) (*n* = 6811)	69.2 (±32.3) (*n* = 892)	66.8 (±28.1) (*n* = 5919)	*

SD = standard deviation; *n* = number of puppies included in the analysis; LBW = low-birth-weight puppies; NBW = normal-birth-weight puppies; GR x–y = growth rate between Day y and Day x, calculated as [(weight at Day y − weight at Day x) ÷ weight at Day x] × 100. ^1^ one-way ANOVA result: *** for *p* < 0.001, ** for *p* < 0.01 and * for *p* < 0.05.

**Table 2:** Thresholds of growth rates discriminating for mortality over the first two months of life as identified through CART analysis.

Birth-Weight Category	Growth Rate	*n*	Growth-Rate Threshold, % (CART Analysis)	Mortality Over the 2 First Months of Age		
				For All Puppies	For Puppies with Growth Rate below the Threshold	For Puppies with Growth Rate Equal or Greater than the Threshold
LBW	GR 0–1	975	−1.8	21%	36% (*n* = 359)	12% (*n* = 616)
	GR 1–2	898	−2.6	17%	53% (*n* = 131)	11% (*n* = 767)
	GR 0–2	1038	−0.4	17%	37% (*n* = 284)	9.2% (*n* = 754)
	GR 2–7	818	31	6.8%	28% (*n* = 106)	3.7% (*n* = 712)
	GR 0–7	892	31	6.7%	25% (*n* = 117)	4% (*n* = 775)
NBW	GR 0–1	5528	−4.1	5.4%	14% (*n* = 1002)	3.6% (*n* = 4526)
	GR 1–2	5350	−7.1	4.5%	39% (*n* = 137)	3.6% (*n* = 5213)
	GR 0–2	6351	−8.7	4.5%	23% (*n* = 404)	3.2% (*n* = 5947)
	GR 2–7	5519	18	1.9%	13% (*n* = 156)	1.6% (*n* = 5363)
	GR 0–7	5919	26	2%	6.3% (*n* = 474)	1.7% (*n* = 5445)

*n* = number of puppies included into the analysis; LBW = low-birth-weight puppies; NBW = normal-birth-weight puppies; GR x–y = growth rate between Day y and Day x, calculated as [(weight at Day y − weight at Day x) ÷ weight at Day x] × 100.

**Table 3:** Relative discriminatory power of weight-related puppy mortality risk factors depending on the day of life.

Day 1		Day 2		Day 7	
Variable	Power	Variable	Power	Variable	Power
GR 0–1	100.00	GR 1–2	100.00	BW category	100
BW category	0	BW category	41.27	GR 2–7	66.78
		GR 0–1	0	GR 0–1	18.24
				GR 1–2	0

BW category = birth-weight category (low birth weight or normal birth weight); GR = growth rate.

## Data Availability

Data and scripts are available from the corresponding author on reasonable request.

## Supplementary Materials

The following supporting information can be downloaded at: https://www.mdpi.com/article/10.3390/ani13121928/s1, Table S1: Birth-weight thresholds defining low- and normal-birth-weight puppies by breed, based on the risk of mortality over the two first months of life (CART analysis).
