# Supplementary material for: Relative Impact of Birth Weight and Early Growth on Neonatal Mortality in Puppies
Source: Animals (Basel). 2023 Jun 9;13(12):1928. doi: 10.3390/ani13121928 (PMC10295787; doi:10.3390/ani13121928)
Supplement: Supplementary file 1 [file animals-13-01928-s001.zip › Table S1.pdf]

Table S1. Birth weight thresholds defining low and normal birth weight puppies by breed, based on the risk of mortality over the two first months of life (CART analysis).

Breed	Birth weight threshold (in grams)
Pomeranian	136
West Highland White Terrier	157
Cavalier King Charles Spaniel	179
Beagle	233
Bulldog	262
Golden Retriever	296
Wirehaired Vizsla	329
Beauceron	361
Leonberger	366
Greyhound	395
Braque d'Auvergne	465
Newfoundland	466
Maltese	115.5
Shetland Sheepdog	132.5
Lhasa Apso	141.5
Yorkshire Terrier	144.5
English Cocker Spaniel	157.5
Shih Tzu	159.5
French Bulldog	185.5
Australian Shepherd Dog	239.5
Labrador Retriever	304.5
German Shepherd Dog	403.5
Boxer	435.5
Berner Sennenhund	500.5
